# Risk of Intracranial Hemorrhage Associated With Direct Oral Anticoagulation vs Antiplatelet Therapy: A Systematic Review and Meta-Analysis

**DOI:** 10.1001/jamanetworkopen.2024.49017

**Published:** 2024-12-04

**Authors:** Mark Coyle, Amy Lynch, Meave Higgins, Maria Costello, Conor Judge, Martin O’Donnell, Catriona Reddin

**Affiliations:** 1 Health Research Board Clinical Research Facility, National University of Ireland Galway, Galway, Ireland; 2 Department of Medicine, Galway University Hospital, Galway, Ireland; 3 Wellcome Trust Health Research Board, Irish Clinical Academic Training, Dublin, Ireland

## Abstract

**Importance:**

For patients with atrial fibrillation, clinicians often prescribe antiplatelet therapy rather than oral anticoagulation, which may be related to a concern that direct oral anticoagulants (DOACs) are associated with a higher risk of intracranial bleeding, despite being less effective for stroke prevention.

**Objective:**

To determine whether DOAC therapy, compared with single-agent antiplatelet therapy, was associated with an increased risk of intracranial and major hemorrhage.

**Data Sources:**

A systematic search of PubMed and Embase databases from inception to February 7, 2024, was performed.

**Study Selection:**

Randomized clinical trials that compared DOAC therapy with single-agent antiplatelet therapies were included. Trials with active follow-up of less than 30 days or a sample size less than 200 were excluded.

**Data Extraction and Synthesis:**

The study followed the Preferred Reporting Items for Systematic Reviews and Meta-Analyses reporting guideline. Data were extracted independently by 2 researchers. A random-effects meta-analysis model was used to report pooled treatment effects and 95% CIs.

**Main Outcomes and Measures:**

The primary outcome was occurrence of intracranial hemorrhage.

**Results:**

A total of 9 randomized clinical trials were included (45 494 participants). DOAC therapy was not associated with significantly higher odds of intracranial hemorrhage compared with antiplatelet therapy (0.55% vs 0.48% over a mean trial follow-up of 17.1 months; odds ratio [OR], 1.15; 95% CI, 0.71-1.88), but there was heterogeneity among trials (*I*^2^ = 53.7%). In an analysis by DOAC agent, the respective estimates for intracranial hemorrhage risk were as follows: rivaroxaban, OR, 2.09 (95% CI, 1.20-3.64); dabigatran, OR, 1.00 (95% CI, 0.61-1.64); and apixaban, OR, 0.72 (95% CI, 0.44-1.17). Overall, DOAC therapy was associated with higher odds of major hemorrhage compared with antiplatelet therapy (2.41% vs 1.76% over a mean trial follow-up of 15.5 months; OR, 1.39; 95% CI, 1.07-1.80), with the following estimates by agent: rivaroxaban, OR, 1.91 (95% CI, 1.22-3.00); dabigatran; OR, 1.21 (95% CI, 0.86-1.69); and apixaban, OR, 1.09 (95% CI, 0.73-1.63).

**Conclusions and Relevance:**

In this systematic review and meta-analysis, DOAC therapy was not associated with a significantly higher risk of intracranial hemorrhage compared with antiplatelet therapy, but was associated with a higher risk of major hemorrhage. These findings support the safety of DOAC compared with antiplatelet therapy with respect to risk of ICH and reinforce adherence with current atrial fibrillation guidelines.

## Key Points

**Question:**

Is there a significant difference in risk of intracranial hemorrhage (ICH) among patients taking direct oral anticoagulation (DOAC) compared with antiplatelet therapy?

**Findings:**

This systematic review and meta-analysis of 9 randomized clinical trials including 45 494 participants found no significant difference in risk of ICH among participants taking DOAC vs single-agent antiplatelet therapy.

**Meaning:**

These findings support the safety of DOAC compared with antiplatelet therapy with respect to risk of ICH and reinforce adherence with current atrial fibrillation guidelines.

## Teaser

This systematic review and meta-analysis of 9 randomized clinical trials examines whether direct oral anticoagulation, vs single-agent antiplatelet therapy, is associated with an increased risk of intracranial and major hemorrhage among patients with atrial fibrillation.

## Introduction

Direct oral anticoagulants (DOACs) are recommended for stroke prevention in patients with nonvalvular atrial fibrillation.^1,2^ Among patients with atrial fibrillation at high risk of bleeding, clinicians often prescribe aspirin instead of a DOAC,^3,4^ despite its lower effectiveness for ischemic stroke prevention.^5^ Intracranial hemorrhage (ICH) is the most feared complication of antithrombotic therapy^6^ and is the most relevant safety outcome in patients with atrial fibrillation at increased risk of ischemic stroke. Clinicians may assume that aspirin is associated with a lower risk of ICH, in part related to trials reporting the safety of aspirin among patients after ICH.^7^ Although there is evidence demonstrating a higher bleeding risk associated with vitamin K antagonists compared with antiplatelet therapy,^8,9^ the safety profile of DOACs is different, particularly with respect to ICH risk. In this meta-analysis, we sought to determine whether DOAC therapy was associated with an increased risk of ICH (and major hemorrhage) compared with single-agent antiplatelet therapy.

## Methods

We performed a systematic review and meta-analysis, adhering to the Cochrane Collaboration Guidelines, and reported our findings according to the Preferred Reporting Items for Systematic Reviews and Meta-analyses (PRISMA) reporting guidelines.^10,11^ The meta-analysis was registered with the International Prospective Register of Systematic Reviews (PROSPERO identifier: CRD42024517308). This study did not require institutional review board approval or informed consent because no human participants were involved, in accordance with 45 CFR §46.

### Data Sources and Search Strategy

We systematically searched PubMed and Embase databases from database inception to February 7, 2024. The search terms included are detailed in the eAppendix in Supplement 1. The search strategy was peer-reviewed by an information specialist (not a coauthor of this article). Following removal of duplicates, titles and abstracts were screened by 2 reviewers (M.C. and A.L.) using the Rayann web application.^12^ The reference lists of included trials and other published meta-analyses were also reviewed. Full texts of remaining articles were independently assessed by 2 reviewers (M.C. and A.L.), with eligibility based on predetermined criteria. Disagreements were resolved by consensus, and where a resolution was not reached by discussion, a consensus was reached through a third reviewer (C.R.).

### Eligibility Criteria, Data Extraction, and Measurements

Studies were considered eligible if they (1) were randomized clinical trials, (2) included adults older than 18 years, (3) evaluated DOAC compared with single-agent antiplatelet therapy, (4) reported bleeding events (at least 1 of the following: ICH, major hemorrhage, or gastrointestinal [GI] hemorrhage), (5) included more than 200 participants, and (6) had a minimum follow-up duration of 30 days. Data were extracted independently by 2 authors (M.C. and A.L.) using a standardized predetermined data collection form. We extracted the following data: study characteristics, baseline demographics of participants, description of the intervention (DOAC) and comparator (antiplatelet therapy), and incidence of outcomes, including ICH, major hemorrhage, GI hemorrhage, fatal hemorrhage, ischemic stroke, all stroke, all-cause mortality, and cardiovascular mortality. We did not prespecify a definition for major hemorrhage. Data were compared for inconsistencies and merged into a prefinal dataset, which was checked independently by 2 other reviewers (C.R. and C.J.).

### Outcomes

The primary outcome measure was ICH. The secondary outcome measures were major hemorrhage, fatal hemorrhage, GI hemorrhage, ischemic stroke, and cardiovascular mortality. The reported outcomes of individual trials are shown in eTable 1 in Supplement 1, and the definitions of major hemorrhage and primary outcomes among the trials are outlined in eTable 2 in Supplement 1. Hemorrhagic stroke was not reported in our results as events were represented within ICH outcome events.

### Statistical Analysis

#### Data Synthesis

We calculated the odds ratio (OR) and 95% CI for each outcome of interest from individual studies. Statistical significance was defined as a 95% CI that did not cross the null value of 1. Weighted pooled treatment effects were calculated using a random-effects model. Summary estimates were calculated for subgroups by DOAC agent and overall. The variability across studies due to heterogeneity was estimated with the *I*^2^ statistic. The *I*^2^ statistic describes the percentage of total variation across studies that is due to heterogeneity rather than chance, and a value greater than 50% indicates the presence of substantial heterogeneity.^10^ For outcomes with trials that had zero events (eg, ICH), a fixed value (0.5) was added for the primary analysis. A sensitivity analysis dropping trials with zero events in both groups was performed. One trial had 2 investigational treatment groups with a common control group; to prevent double counting, we split the common control group into 2 equal-sized groups.^10,13^ Publication bias was assessed using a funnel plot (eFigure 1 in Supplement 1). A priori sensitivity analyses were performed by DOAC indication and included trials in which major hemorrhage was defined by the International Society on Thrombosis and Hemostasis (ISTH) definition.^14^

#### Risk of Bias Assessment

We used the Cochrane risk-of-bias tool for randomized trials to assess methodological quality of eligible trials.^15^ Trials were assessed on 5 domains: randomization process, deviations from intended interventions, missing outcome data, measurement of the outcome, and selection of the reported result. Risk of bias assessments were performed independently by 2 reviewers (M.C. and M.H.), and disagreements were resolved by a third reviewer (C.R.). Studies were deemed at high risk of bias overall if 1 or more domains were rated as high, or if multiple domains were judged to have “some concerns in a way that substantially lower confidence in the result.”^15^ Risk of bias summary tables were created.

## Results

Our systematic search of articles published before February 7, 2024, identified 2216 records. Following title and abstract screening, 75 articles were considered potentially relevant. After application of eligibility criteria to full-text review, 9 trials (45 494 participants) were included (eFigure 2 in Supplement 1). Of these, 7 trials were double-blinded, randomized clinical trials,^13,16,17,18,19,20,21^ and 2 trials used a prospective randomized, open-label blinded–end point design.^22,23^ The intervention group was apixaban for 4 trials,^17,20,21,22^ dabigatran for 2 trials,^19,23^ and rivaroxaban for 3 trials.^13,16,18^ Two trials, COMPASS^16^ and EINSTEIN^13^ choice (1 intervention group), evaluated intermediate-dose rivaroxaban. The comparator for all included trials was aspirin (Table). The mean (SD) follow-up duration was 17.2 (10.8) months. The mean (SD) age of participants was 67.5 (4.6) years. The outcomes reported by individual trials are reported in eTable 1 in Supplement 1. Overall, the risk of bias in the included studies was low (eFigure 3 in Supplement 1). There was no evidence of publication bias for the primary outcome (eFigure 1 in Supplement 1).

**Table.** Trial Characteristics

Source	Participants, No.	Study population	Mean age, y	Intervention group	Control group	Follow-up duration, mo
Hart et al,^18^ 2018	7213	Embolic stroke of undetermined source within 7 d to 6 mo (nonlacunar stroke, with no significant carotid disease, atrial fibrillation, left ventricle thrombus or mechanical valve, or severe mitral stenosis)	66.9	Rivaroxaban 15 mg	Aspirin 100 mg	11
Diener et al,^19^ 2019	5390	Embolic stroke of undetermined source; age >60 y (nonlacunar ischemic stroke with no significant carotid disease, atrial fibrillation or left ventricle thrombus)	64	Dabigatran 150 mg twice daily or 110 mg twice daily	Aspirin 100 mg	19
Weitz et al,^13^ 2017	3365	Venous thromboembolism, treated for 6-12 mo with oral anticoagulation	58.4	Rivaroxaban 20 mg or rivaroxaban 10 mg	Aspirin 100 mg	12
Butcher et al,^23^ 2020	305	Transient ischemic attack or minor ischemic stroke (National Institutes of Health Stroke Scale score <9)	66.6	Dabigatran 150 mg twice daily or 110 mg twice daily	Aspirin 81 mg	1
Connolly et al,^20^ 2011	5599	Documented history of atrial fibrillation in the last 6 mo with at least 1 additional risk factor for stroke	70	Apixaban 5 mg twice daily	Aspirin 81-324 mg	13.2
Healey et al,^17^ 2024	4012	Subclinical atrial fibrillation detected by a cardiac monitor or implanted cardiac device, lasting >6 min but <24 h; CHADS_2_-VASC score ≥ to 3	76.8	Apixaban 5 mg twice daily	Aspirin 81 mg	42
Eikelboom et al,^16^ 2017	18 243	Stable cardiovascular disease (coronary disease and or peripheral arterial disease)	68.2	Rivaroxaban 5 mg twice daily	Aspirin 100 mg	23
Kamel et al,^21^ 2024	1015	Patients aged >45 y with cryptogenic stroke and evidence of atrial cardiopathy	68	Apixaban 5 mg twice daily	Aspirin 81 mg	21.6
Geisler et al,^22^ 2024	352	Patients with embolic stroke of undetermined source with ≥1 risk factor for atrial fibrillation or a patent foramen ovale	68.5	Apixaban 5 mg twice daily	Aspirin 100 mg	12

Abbreviation: CHADS_2_-VASC, congestive heart failure, hypertension, age ≥75 years, diabetes, stroke or transient ischemic attack, vascular disease, age 65 to 74 years, and sex category.

### Intracranial Hemorrhage

**Figure 1.**
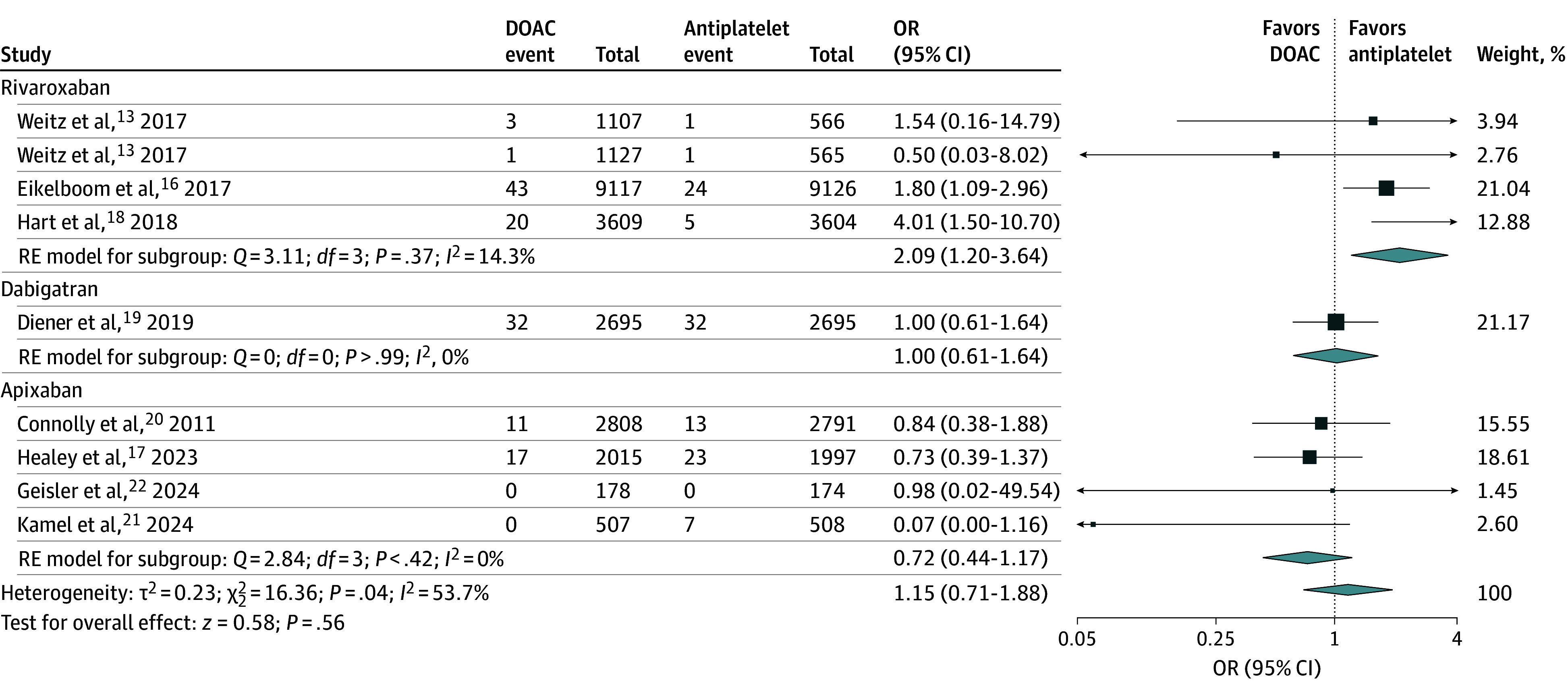
Association of Direct Oral Anticoagulation (DOAC) vs Antiplatelet Therapy With Symptomatic Intracranial Hemorrhage Forest plot shows the association of DOAC vs antiplatelet therapy with symptomatic intracranial hemorrhage. The squares and horizontal lines represent the mean values and 95% CIs of the effect sizes, and the area of the squares reflects the weight of the studies. The combined effect estimates appear as diamonds, and the vertical dashed line represents the line of no effect. OR indicates odds ratio; and RE, random effects.

Among 8 trials (45 189 participants), there were 233 ICH events during follow-up, including 127 in the DOAC group and 106 in the antiplatelet group.^13,16,17,18,19,20,21,22^ DOAC therapy was not associated with significantly higher odds of ICH compared with antiplatelet therapy (0.55% vs 0.48% over a mean trial follow-up of 17.1 months; OR, 1.15; 95% CI, 0.71-1.88) (Figure 1). There was heterogeneity among DOAC agents (overall *I*^2^ = 53.7%). In an analysis by DOAC agent, the respective estimates for ICH risk were as follows: rivaroxaban, OR, 2.09 (95% CI, 1.20-3.64); dabigatran, OR, 1.00 (95% CI, 0.61-1.64); and apixaban, OR, 0.72 (95% CI, 0.44-1.17).

### Major Hemorrhage

**Figure 2.**
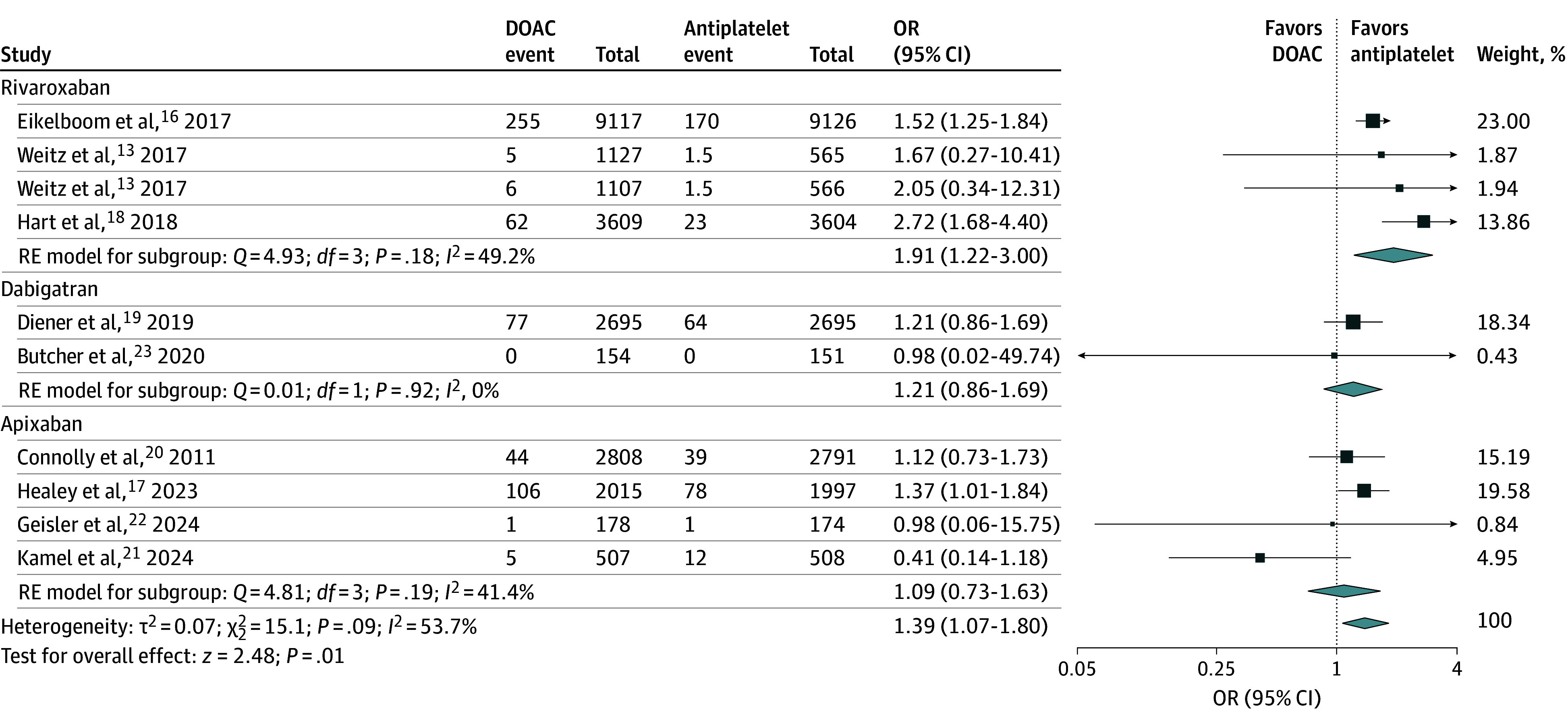
Association of Direct Oral Anticoagulation (DOAC) vs Antiplatelet Therapy and Major Hemorrhage Forest plot shows the association of DOAC therapy vs antiplatelet therapy with major hemorrhage events. The squares and horizontal lines represent the mean values and 95% CIs of the effect sizes, and the area of the squares reflects the weight of the studies. The combined effect estimates appear as diamonds, and the vertical dashed line represents the line of no effect. OR indicates odds ratio; and RE, random effects.

Among 9 trials (45 494 participants), there were 951 major hemorrhage events in total; 561 occurred in the DOAC group and 390 in the antiplatelet group. DOAC therapy was associated with significantly higher odds of major hemorrhage compared with antiplatelet therapy (2.41% vs 1.76% over a mean trial follow-up of 15.5 months; OR, 1.39; 95% CI, 1.07-1.80). There was heterogeneity among DOAC agents (overall *I*^2^ = 53.7%). In an analysis by DOAC agent, the respective estimates for major hemorrhage risk were as follows: rivaroxaban, OR, 1.91 (95% CI, 1.22-3.00); dabigatran, OR, 1.21 (95% CI, 0.86-1.69); and apixaban, OR, 1.09 (95% CI, 0.73-1.63) (Figure 2). The definition for major hemorrhage differed among trials and is outlined in eTable 2 in Supplement 1. A sensitivity analysis limited to major hemorrhage defined by ISTH definition did not materially alter the findings (eFigure 4 in Supplement 1).

**Figure 3.**
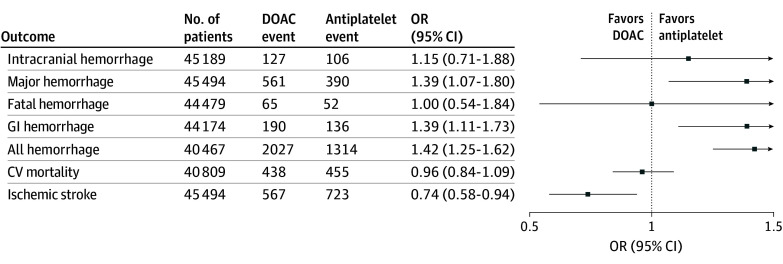
Association of Direct Oral Anticoagulation (DOAC) vs Antiplatelet Therapy With Hemorrhage Outcomes Forest plot shows the association of DOAC therapy vs antiplatelet therapy with outcome events. The squares and horizontal lines represent the mean values and 95% CIs of the effect sizes, and the area of the squares reflects the weight of the studies. The vertical dashed line represents the line of no effect. CV indicates cardiovascular; GI, gastrointestinal; and OR, odds ratio.

**Figure 4.**
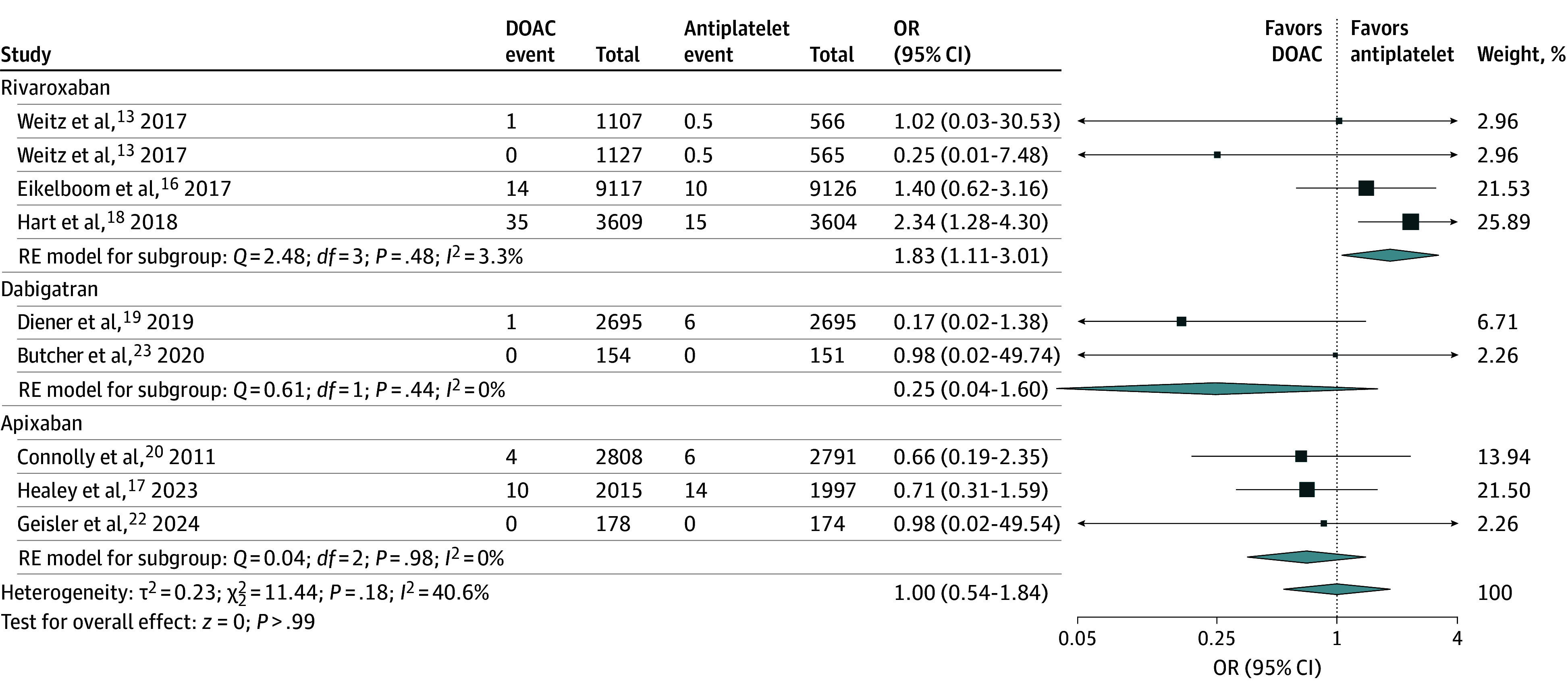
Association of Direct Oral Anticoagulation (DOAC) vs Antiplatelet Therapy and Fatal Hemorrhage Forest plot shows the association of DOAC therapy vs antiplatelet therapy with fatal hemorrhage. The squares and horizontal lines represent the mean values and 95% CIs of the effect sizes, and the area of the squares reflects the weight of the studies. The combined effect estimates appear as diamonds, and the vertical dashed line represents the line of no effect. OR indicates odds ratio; and RE, random effects.

Among 7 trials (44 174 participants), there were 326 GI hemorrhages in total, including 190 cases in the DOAC group and 136 cases in the antiplatelet group.^13,16,17,18,19,20,22^ DOAC therapy was associated with significantly higher odds of GI hemorrhage compared with antiplatelet therapy (0.84% vs 0.63% over a mean trial follow-up of 16.5 months; OR, 1.39; 95% CI, 1.11-1.73) (Figure 3 and eFigure 5 in Supplement 1). Eight trials (44 479 participants)^13,16,17,18,19,20,22,23^ reported fatal hemorrhage. There were 117 fatal hemorrhages in total, including 65 in the DOAC group and 52 in the antiplatelet group. There was no significant association between DOAC use and fatal hemorrhage (0.28% vs 0.24% over a mean trial follow-up of 14.8 months, OR, 1.00; 95% CI, 0.54-1.84) (Figure 4). Seven trials (40 467 participants)^13,16,18,19,20,22,23^ reported all hemorrhage as an outcome, including 2027 in the DOAC group and 1314 in the antiplatelet group. DOAC therapy was associated with significantly higher odds of all hemorrhage compared with antiplatelet therapy (9.7% vs 6.7% over a mean trial follow-up of 11.4 months; OR, 1.42; 95% CI, 1.25-1.62) (Figure 3 and eFigure 6 in Supplement 1).

### Ischemic Stroke and Cardiovascular Death

Among 9 trials (45 494 participants),^13,16,17,18,19,20,21,22,23^ there were 1290 ischemic stroke events; 567 occurred in the DOAC group, and 723 in the antiplatelet group. DOAC therapy was associated with significantly lower odds of ischemic stroke compared with antiplatelet therapy (2.43% vs 3.26% over a mean trial follow-up of 15.5 months; OR, 0.74; 95% CI, 0.58-0.94) (Figure 3 and eFigure 7 in Supplement 1). Among 6 trials (40 809 participants),^16,17,18,19,20,22^ there were 893 cardiovascular death events: 438 in the DOAC group and 455 in the antiplatelet group. DOAC therapy was not associated with significantly lower odds of cardiovascular death compared with antiplatelet therapy (2.14% vs 2.23% over a mean trial follow-up of 20.0 months; OR, 0.96; 95% CI, 0.84-1.09) (Figure 3 and eFigure 8 in Supplement 1). Sensitivity analysis including excluding trials with zero outcomes in both treatment groups for the primary outcome did not materially alter the findings for the primary outcome (eFigure 9 in Supplement 1).

## Discussion

In this systematic review and meta-analysis of randomized clinical trials comparing DOAC with single-agent antiplatelet therapy (8 trials included for the primary outcome; 45 189 participants), we found no significant increase in risk of ICH. However, our exploration of heterogeneity among trials suggested different effects by agent, whereby rivaroxaban, but not apixaban or dabigatran, was associated with a significantly increased risk of ICH. However, other differences among trials (eg, population) preclude definitive conclusions on differences among DOAC agents. DOAC therapy was associated with a significantly increased risk of major hemorrhage, with similar heterogeneity of effect among agents. Our findings do not support the contention that aspirin is associated with a lower risk of ICH, most convincingly for apixaban.

A major advantage of DOACs over vitamin K antagonists is the substantially lower risk of ICH, reported in all major clinical trials comparing agents among patients with atrial fibrillation.^24^ Until relatively recently, few clinical trials compared DOACs with single-agent antiplatelet therapy, and a prior meta-analysis addressed a similar research question.^25^ Since that analysis in 2021,^25^ there have been 3 additional randomized clinical trials reported (ARTESIA,^17^ ARCADIA,^21^ and ATTICUS^22^), which are included in our study. Accordingly, we are able to report more robust estimates due to larger sample size and additional events. However, despite the increase in sample size, the 95% CIs for most of our estimates are relatively wide, precluding definitive conclusions.

The most common indication for oral anticoagulation is atrial fibrillation.^26^ Registry studies report that a substantial proportion of patients with atrial fibrillation are prescribed single-antiplatelet agents (usually aspirin) rather than DOACs.^3^ One reason that may influence decision-making is concern for major bleeding, particularly ICH, with the assumption that aspirin is safer than DOAC owing to limited data and prior experience with vitamin K antagonists.^2^ Findings from the RESTART trial likely add further evidence of the safety of aspirin, as it did not increase the risk of recurrent ICH among patients with a prior ICH.^7^ A barrier to prescription is perceived risk of hemorrhage,^6^ with studies reporting that clinicians are more risk-averse than patients.^27^ Our findings support the use of a DOAC, particularly apixaban, in preference to aspirin among patients with atrial fibrillation considered at high risk of major hemorrhage due to the safety data reported and efficacy for ischemic stroke prevention. Our data suggest, but do not prove, differences in comparative risk of ICH among agents, which has also been reported in previous observational evidence and indirect comparisons from randomized clinical trials.^28,29,30^ However, we are unable to report on a head-to-head comparison between agents, so our findings need to be interpreted with caution as differences may be attributable to other differences among trials, including population and dosing regimens.^31,32^

### Limitations

This study has several limitations. First, intervention groups consisted of a combination of different DOAC agents. We report overall estimates and estimates by DOAC agents. The comparator group was aspirin for all trials. Second, the population enrolled differed between trials (eg, atrial fibrillation vs embolic stroke of undetermined source). Although the absolute risk of bleeding events may differ between populations, the relative risk estimate for treatment effect is expected to be consistent among populations.^33^ Third, definitions of major hemorrhage varied among studies. We have outlined the definition of major hemorrhage adopted by each trial in eTable 2 in Supplement 1 for clarity, and conducted an analysis limited to ISTH definition of major hemorrhage. The definition of major hemorrhage used was unavailable for one trial.^23^ Fourth, there were low event rates for some outcomes (eg, fatal hemorrhage), with consequently imprecise summary estimates. This meta-analysis expands on previous work by including 3 recent large randomized clinical trials and provides the most up-to-date synthesis of evidence to address this clinical question.

## Conclusions

In conclusion, this study found no evidence of a significant increase in ICH risk associated with DOAC therapy compared with antiplatelet therapy. DOAC use was associated with significantly lower rates of ischemic stroke but higher rates of major hemorrhage. This meta-analysis supports the safety of DOAC compared with antiplatelet therapy with regard to risk of ICH.

## Notes

Supplement 1. **eAppendix**. Search strategy **eTable 1.** Reported outcomes of individual trials **eTable 2.** Definition of major haemorrhage of individual trials **eFigure 1.** Funnel plot **eFigure 2.** PRISMA flow diagram **eFigure 3.** Risk of bias 2 assessment **eFigure 4.** Association of DOAC compared to antiplatelet therapy with ISTH defined major haemorrhage **eFigure 5.** Association of DOAC compared to antiplatelet therapy with GI haemorrhage **eFigure 6.** Association of DOAC compared to antiplatelet therapy with all haemorrhage **eFigure 7.** Association of DOAC compared to antiplatelet therapy with ischaemic stroke **eFigure 8.** Association of DOAC compared to antiplatelet therapy with CV death **eFigure 9.** Association of DOAC compared to antiplatelet therapy with intracranial haemorrhage, sensitivity analysis excluding trials with zero events

Supplement 2.
Data Sharing Statement
