# Supplementary material for: Risk of Intracranial Hemorrhage Associated With Direct Oral Anticoagulation vs Antiplatelet Therapy: A Systematic Review and Meta-Analysis
Source: JAMA Netw Open. 2024 Dec 4;7(12):e2449017. doi: 10.1001/jamanetworkopen.2024.49017 (PMC11618459; doi:10.1001/jamanetworkopen.2024.49017)
Supplement: Supplement 1. — eAppendix. Search strategy eTable 1. Reported outcomes of individual trials eTable 2. Definition of major haemorrhage of individual trials eFigure 1. Funnel plot eFigure 2. PRISMA flow diagram eFigure 3. Risk of bias 2 assessment eFigure 4. Association of DOAC compared to antiplatelet therapy with ISTH defined major haemorrhage eFigure 5. Association of DOAC compared to antiplatelet therapy with GI haemorrhage eFigure 6. Association of DOAC compared to antiplatelet therapy with all haemorrhage eFigure 7. Association of DOAC compared to antiplatelet therapy with ischaemic stroke eFigure 8. Association of DOAC compared to antiplatelet therapy with CV death eFigure 9. Association of DOAC compared to antiplatelet therapy with intracranial haemorrhage, sensitivity analysis excluding trials with zero events [file jamanetwopen-e2449017-s001.pdf]

Supplemental Online Content

Coyle M, Lynch A, Higgins M, et al. Risk of intracranial hemorrhage associated with direct oral anticoagulation vs antiplatelet therapy: a systematic review and meta-analysis. *JAMA Netw Open*. 2024;7(12):e2449017. doi:10.1001/jamanetworkopen.2024.49017

eAppendix. Search strategy
eTable 1. Reported outcomes of individual trials
eTable 2. Definition of major haemorrhage of individual trials
eFigure 1. Funnel plot
eFigure 2. PRISMA flow diagram
eFigure 3. Risk of bias 2 assessment
eFigure 4. Association of DOAC compared to antiplatelet therapy with ISTH defined major haemorrhage
eFigure 5. Association of DOAC compared to antiplatelet therapy with GI haemorrhage
eFigure 6. Association of DOAC compared to antiplatelet therapy with all haemorrhage
eFigure 7. Association of DOAC compared to antiplatelet therapy with ischaemic stroke
eFigure 8. Association of DOAC compared to antiplatelet therapy with CV death
eFigure 9. Association of DOAC compared to antiplatelet therapy with intracranial haemorrhage, sensitivity analysis excluding trials with zero events

This supplemental material has been provided by the authors to give readers additional information about their work.

© 2024 Coyle M et al. *JAMA Network Open.*

eAppendix. Search strategy

Studies were searched through February 7th 2024.

Search terms were as follows:

Rivaroxaban *OR* Edoxaban *OR* Apixaban *OR* Dabigatran
AND
Aspirin *OR* Clopidogrel *OR* Triflusal *OR* Ticagrelor *OR* Prasugrel *OR* Dipyridamole *OR* Cilostazol
AND
Randomized controlled trial *OR* Randomized controlled trial *OR* Controlled clinical trial *OR* Randomized *OR* Randomized OR placebo *OR* Drug therapy *OR* Randomly *OR* Trial *OR* Groups
NOT
Animals *NOT* Humans

The search was conducted through Pubmed and EMBASE.

© 2024 Coyle M et al. *JAMA Network Open.*

eTable 1. Reported outcomes of individual trials

Trial	Outcome						
	**Intracranial haemorrhage**	**Major haemorrhage**	**Fatal haemorrhage**	**Gastrointestinal haemorrhage**	**All haemorrhage**	**Ischaemic stroke**	**Cardiovascular mortality**
ATTICUS 2023	√	√	√	√	√	√	√
ARCADIA 2024	√	√				√	
ARTESIA 2023	√	√	√	√		√	√
DATAS II 2020*		√	√		√	√	√
RE-SPECT ESUS 2019	√	√	√	√	√	√	√
NAVIGATE ESUS 2018	√	√	√	√	√	√	√
EINSTEIN CHOICE 2017	√	√	√	√	√	√	
COMPASS 2017	√	√	√	√	√	√	√
AVERROES 2011	√	√	√	√	√	√	√

*Symptomatic haemorrhagic transformation was not considered as primary intracranial haemorrhage

© 2024 Coyle M et al. *JAMA Network Open.*

eTable 2. Definition of major haemorrhage of individual trials

Trial	Major haemorrhage definition
ATTICUS 2023	ISTH definition for major bleeding*
ARCADIA 2024	Clinically overt bleeding accompanied by a 2-g/dL or greater decrease in the hemoglobin level during a 24-hour period, transfusion of 2 units or more of whole blood or red blood cells, involvement of a critical non-intracranial site (intraspinal, intraocular, pericardial, intra-articular, intramuscular with compartment syndrome, or retroperitoneal), or death.
ARTESIA 2023	ISTH definition for major bleeding*
DATAS II 2020	Definition not reported
RE-SPECT ESUS 2019	ISTH definition for major bleeding*
NAVIGATE ESUS 2018	ISTH definition for major bleeding*
EINSTEIN CHOICE 2017	ISTH definition for major bleeding*
COMPASS 2017	All bleeding leading to presentation to an acute care facility or hospital
AVERROES 2011	Clinically overt bleeding accompanied by one or more of the following: a decrease in the hemoglobin level of 2 g per deciliter or more over a 24-hour period, transfusion of 2 or more units of packed red cells, bleeding at a critical site (intracranial, intraspinal, intraocular, pericardial, intraarticular, intramuscular with compartment syndrome, or retroperitoneal), or fatal bleeding

*ISTH definition = Fatal bleeding and/or symptomatic bleeding in a critical area or organ, such as intracranial, intraspinal, intraocular, retroperitoneal, intra-articular or pericardial, or intramuscular with compartment syndrome, and/or bleeding causing a fall in haemoglobin level of 20 gL^{-1} (1.24 $mmolL^{-1}$) or more, or leading to transfusion of two or more units of whole blood or red cells[15]

© 2024 Coyle M et al. *JAMA Network Open.*

eFigure 1. Funnel plot

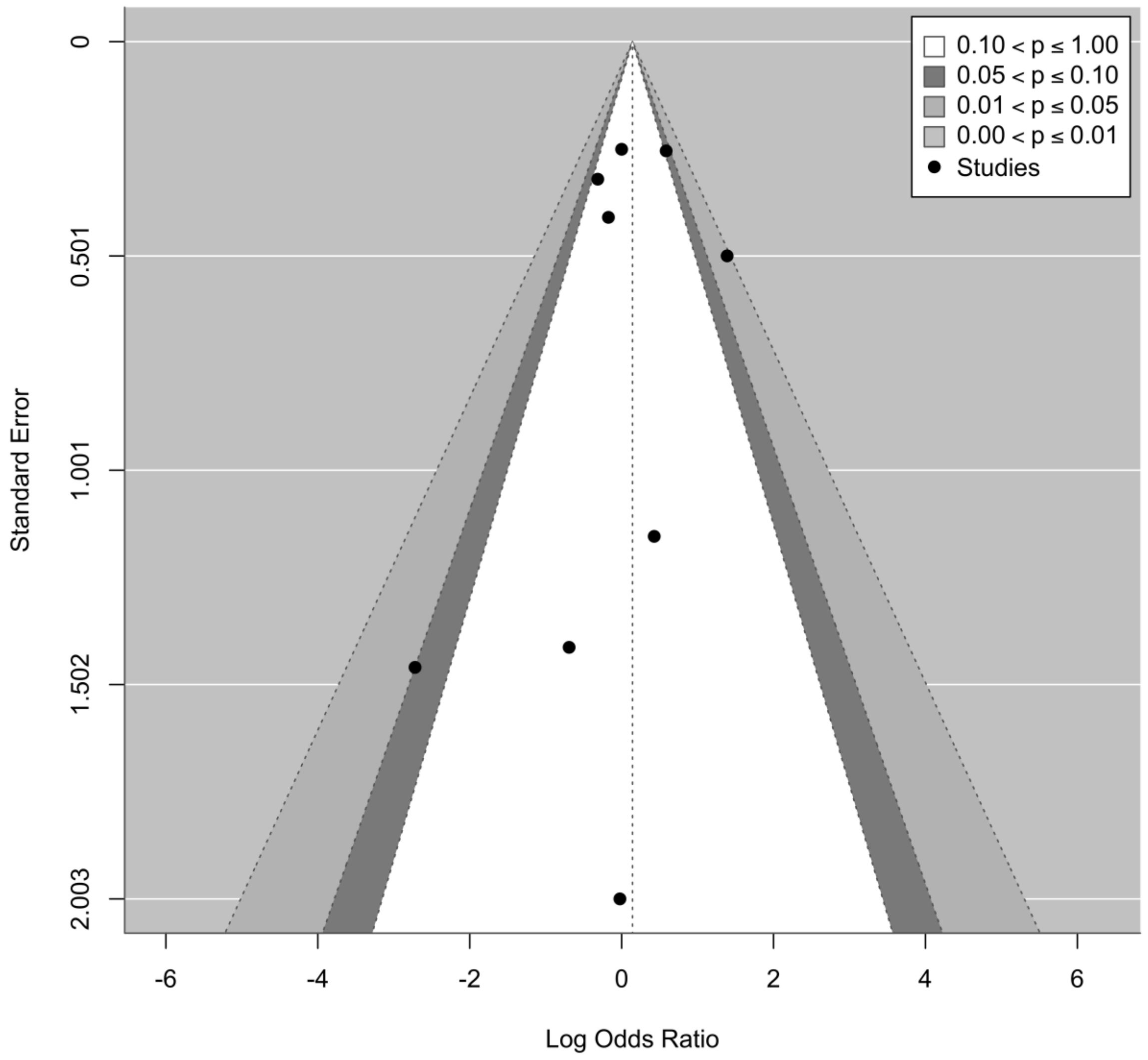

eFigure 1 – Contour enhanced funnel plot for the primary outcome; intracranial haemorrhage. Different levels of statistical significance for studies are indicated by the shaded regions, detailed within the figure. The grey vertical line represents the summary estimate for the association of DOAC therapy compared to antiplatelet therapy with intracranial haemorrhage outcome.

© 2024 Coyle M et al. *JAMA Network Open.*

eFigure 2. PRISMA flow diagram

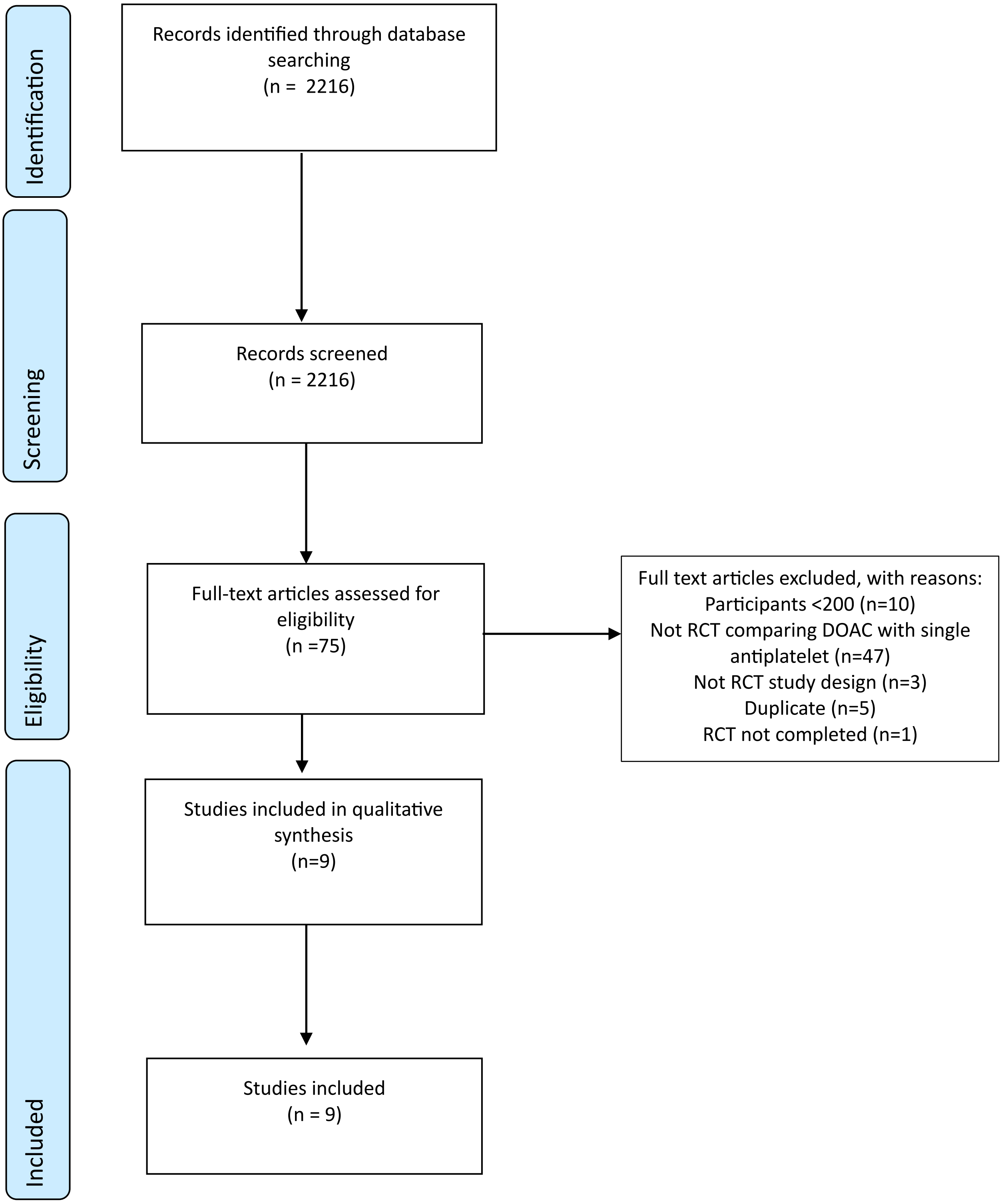

© 2024 Coyle M et al. *JAMA Network Open.*

eFigure 3. Risk of bias 2 assessment

Study ID	Experimental	Comparator	D1	D2	D3	D4	D5	Overall
NAVIGATE ESUS	Rivaroxaban	Aspirin	+	+	+	+	+	+
RESPECT ESUS	Dabigatran	Aspirin	+	+	+	+	+	+
EINSTEIN CHOICE	Rivaroxaban	Aspirin	+	+	+	+	+	+
DATAS II	Dabigatran	Aspirin	+	!	+	+	+	+
AVERROES	Apixaban	Aspirin	+	+	+	+	+	+
ARTESIA	Apixaban	Aspirin	+	+	+	+	+	+
COMPASS	Rivaroxaban	Aspirin	+	+	+	+	+	+
ARCADIA	Apixaban	Aspirin	+	+	+	+	+	+
ATTICUS	Apixaban	Aspirin	+	+	+	+	+	+

+ Low risk
! Some concerns
- High risk

D1 Randomisation process
D2 Deviations from the intended interventions
D3 Missing outcome data
D4 Measurement of the outcome
D5 Selection of the reported result

© 2024 Coyle M et al. *JAMA Network Open.*

eFigure 4. Association of DOAC compared to antiplatelet therapy with ISTH defined major haemorrhage

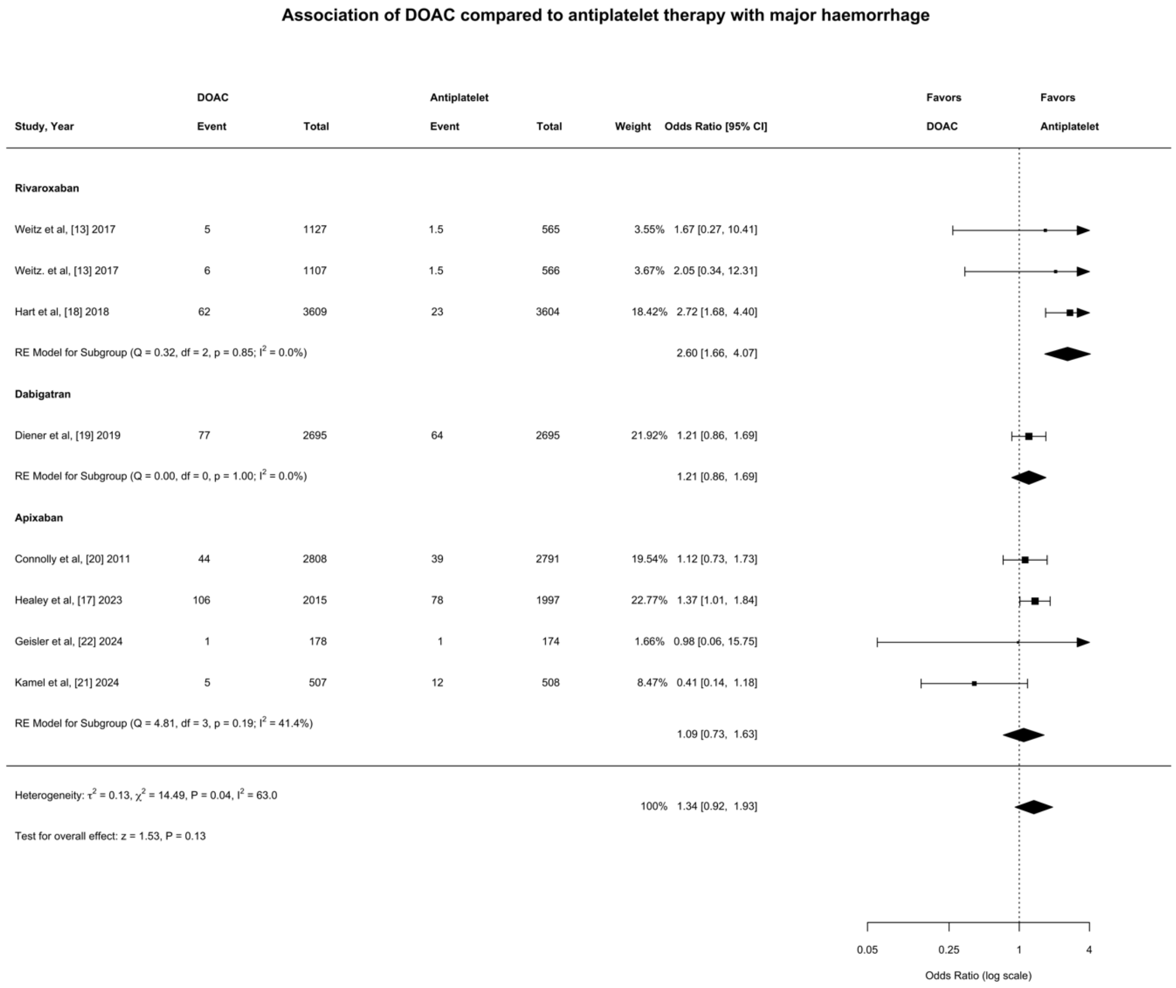

eFigure 4 – Forest plot demonstrating the association of direct oral anticoagulant therapy compared to antiplatelet therapy with major haemorrhage according to the ISTH definition for major haemorrhage. The squares and bars represent the mean values and 95% confidence intervals of the effect sizes, while the area of the squares reflects the weight of the studies. The combined effects appear as diamonds and the vertical dashed line represents the line of no effect.
DOAC- Direct oral anticoagulation, CI-Confidence Interval, ISTH-International Society on Thrombosis and Haemostasis.

© 2024 Coyle M et al. *JAMA Network Open.*

eFigure 5. Association of DOAC compared to antiplatelet therapy with GI haemorrhage

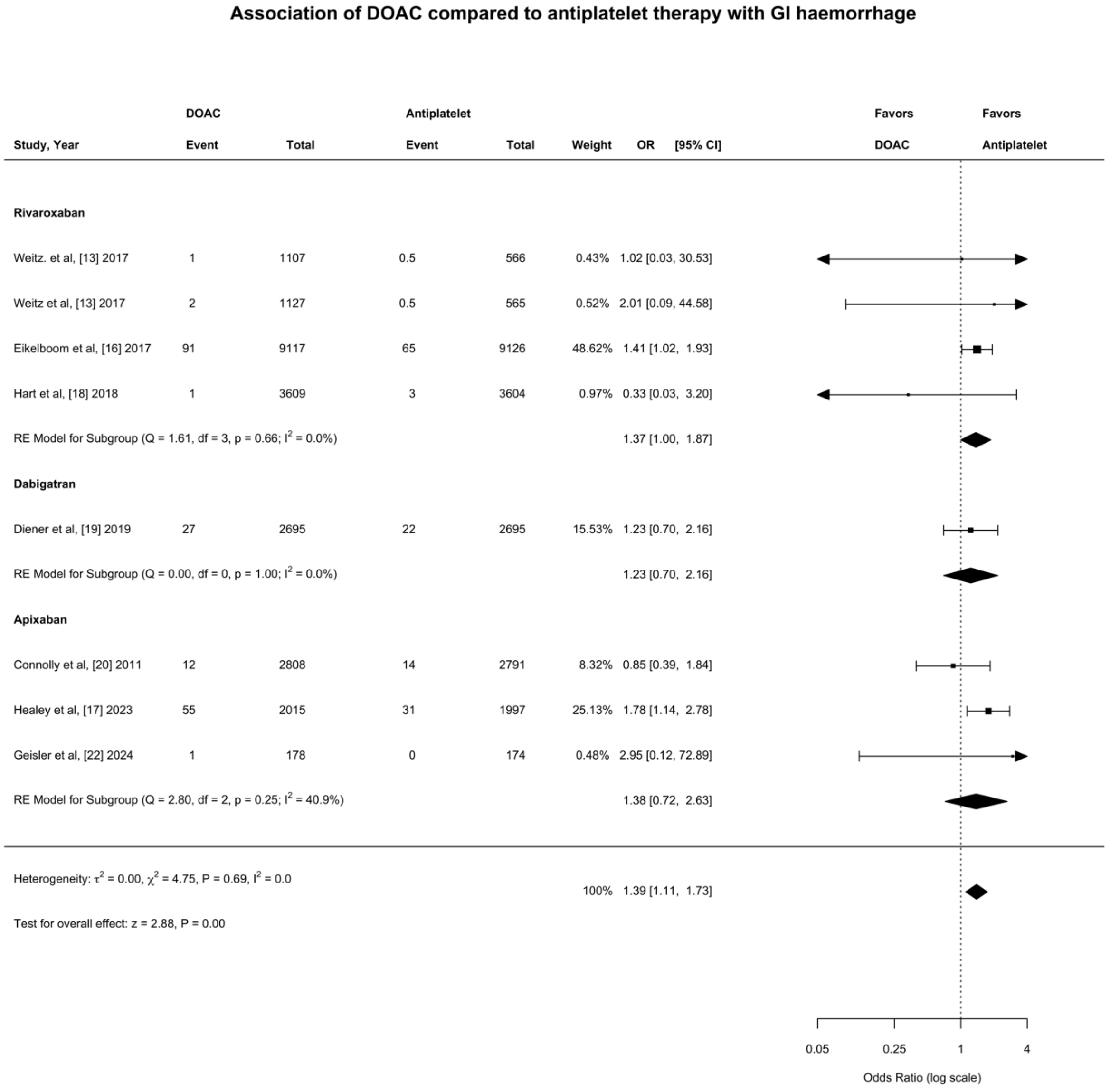

eFigure 5 – Forest plot demonstrating the association of direct oral anticoagulant therapy compared to antiplatelet therapy with GI haemorrhage events. The squares and bars represent the mean values and 95% confidence intervals of the effect sizes, while the area of the squares reflects the weight of the studies. The combined effects appear as diamonds and the vertical dashed line represents the line of no effect.
DOAC- Direct oral anticoagulation, CI-Confidence Interval, GI-gastrointestinal.

© 2024 Coyle M et al. *JAMA Network Open.*

eFigure 6. Association of DOAC compared to antiplatelet therapy with all haemorrhage

Association of DOAC compared to antiplatelet therapy with all haemorrhage

Study, Year	DOAC Event	DOAC Total	Antiplatelet Event	Antiplatelet Total	Weight	OR [95% CI]
**Rivaroxaban**						
Weitz. et al, [13] 2017	196	1107	72.5	566	11.95%	1.46 [1.09, 1.96]
Weitz et al, [13] 2017	160	1127	72.5	565	11.61%	1.12 [0.83, 1.51]
Eikelboom et al, [16] 2017	996	9117	673	9126	25.48%	1.54 [1.39, 1.71]
Hart et al, [18] 2018	180	3609	102	3604	14.36%	1.80 [1.41, 2.31]
RE Model for Subgroup (Q = 5.89, df = 3, p = 0.12; I^2 = 49.8%)						1.49 [1.28, 1.75]
**Dabigatran**						
Diener et al, [19] 2019	147	2695	105	2695	13.82%	1.42 [1.10, 1.84]
Butcher et al, [23] 2020	15	154	6	151	1.66%	2.61 [0.98, 6.91]
RE Model for Subgroup (Q = 1.39, df = 1, p = 0.24; I^2 = 27.9%)						1.59 [1.00, 2.53]
**Apixaban**						
Connolly et al, [20] 2011	328	2808	276	2791	19.94%	1.21 [1.02, 1.43]
Geisler et al, [22] 2024	5	178	7	174	1.18%	0.69 [0.21, 2.22]
RE Model for Subgroup (Q = 0.86, df = 1, p = 0.35; I^2 = 0.0%)						1.19 [1.01, 1.41]
Heterogeneity: τ^2 = 0.01, χ^2 = 14.67, P = 0.04, I^2 = 50.0					100%	1.42 [1.25, 1.62]
Test for overall effect: z = 5.33, P = 0.00						

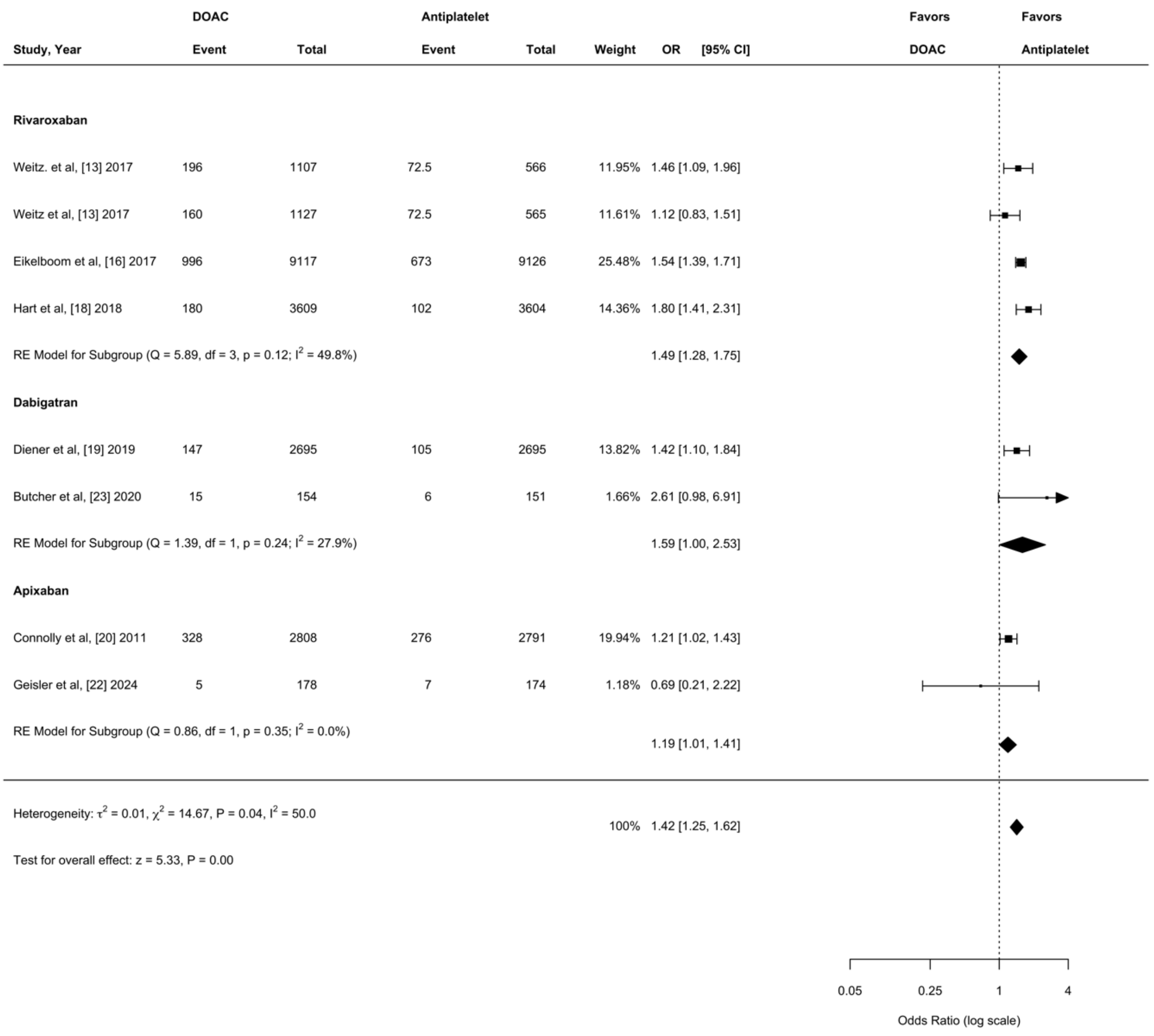

eFigure 6 – Forest plot demonstrating the association of direct oral anticoagulant therapy compared to antiplatelet therapy with all haemorrhage. The squares and bars represent the mean values and 95% confidence intervals of the effect sizes, while the area of the squares reflects the weight of the studies. The combined effects appear as diamonds and the vertical dashed line represents the line of no effect. DOAC-Direct oral anticoagulation, CI-Confidence Interval.

© 2024 Coyle M et al. *JAMA Network Open.*

eFigure 7. Association of DOAC compared to antiplatelet therapy with ischaemic stroke

Association of DOAC compared to antiplatelet therapy with ischaemic stroke

Study, Year	DOAC Event	DOAC Total	Antiplatelet Event	Antiplatelet Total	Weight	OR [95% CI]
**Rivaroxaban**						
Weitz. et al, [13] 2017	2	1107	1	566	0.99%	1.02 [0.09, 11.30]
Weitz et al, [13] 2017	4	1127	1	565	1.17%	2.01 [0.22, 18.02]
Eikelboom et al, [16] 2017	91	9117	132	9126	15.58%	0.69 [0.53, 0.90]
Hart et al, [18] 2018	158	3609	156	3604	16.48%	1.01 [0.81, 1.27]
RE Model for Subgroup ($Q = 5.26$, $df = 3$, $p = 0.15$; $I^2 = 52.8\%$)						0.86 [0.61, 1.23]
**Dabigatran**						
Diener et al, [19] 2019	172	2695	203	2695	16.80%	0.84 [0.68, 1.03]
Butcher et al, [23] 2020	9	154	14	151	5.62%	0.61 [0.25, 1.45]
RE Model for Subgroup ($Q = 0.49$, $df = 1$, $p = 0.48$; $I^2 = 0.0\%$)						0.82 [0.67, 1.01]
**Apixaban**						
Connolly et al, [20] 2011	35	2808	93	2791	12.86%	0.37 [0.25, 0.54]
Healey et al, [17] 2023	45	2015	71	1997	13.15%	0.62 [0.42, 0.90]
Geisler et al, [22] 2024	11	178	12	174	5.84%	0.89 [0.38, 2.07]
Kamel et al, [21] 2024	40	507	40	508	11.51%	1.00 [0.63, 1.58]
RE Model for Subgroup ($Q = 11.79$, $df = 3$, $p = 0.01$; $I^2 = 74.0\%$)						0.64 [0.40, 1.03]
Heterogeneity: $\tau^2 = 0.08$, $\chi^2 = 24.40$, $P = 0.00$, $I^2 = 68.3$					100%	0.74 [0.58, 0.94]
Test for overall effect: $z = -2.44$, $P = 0.01$						

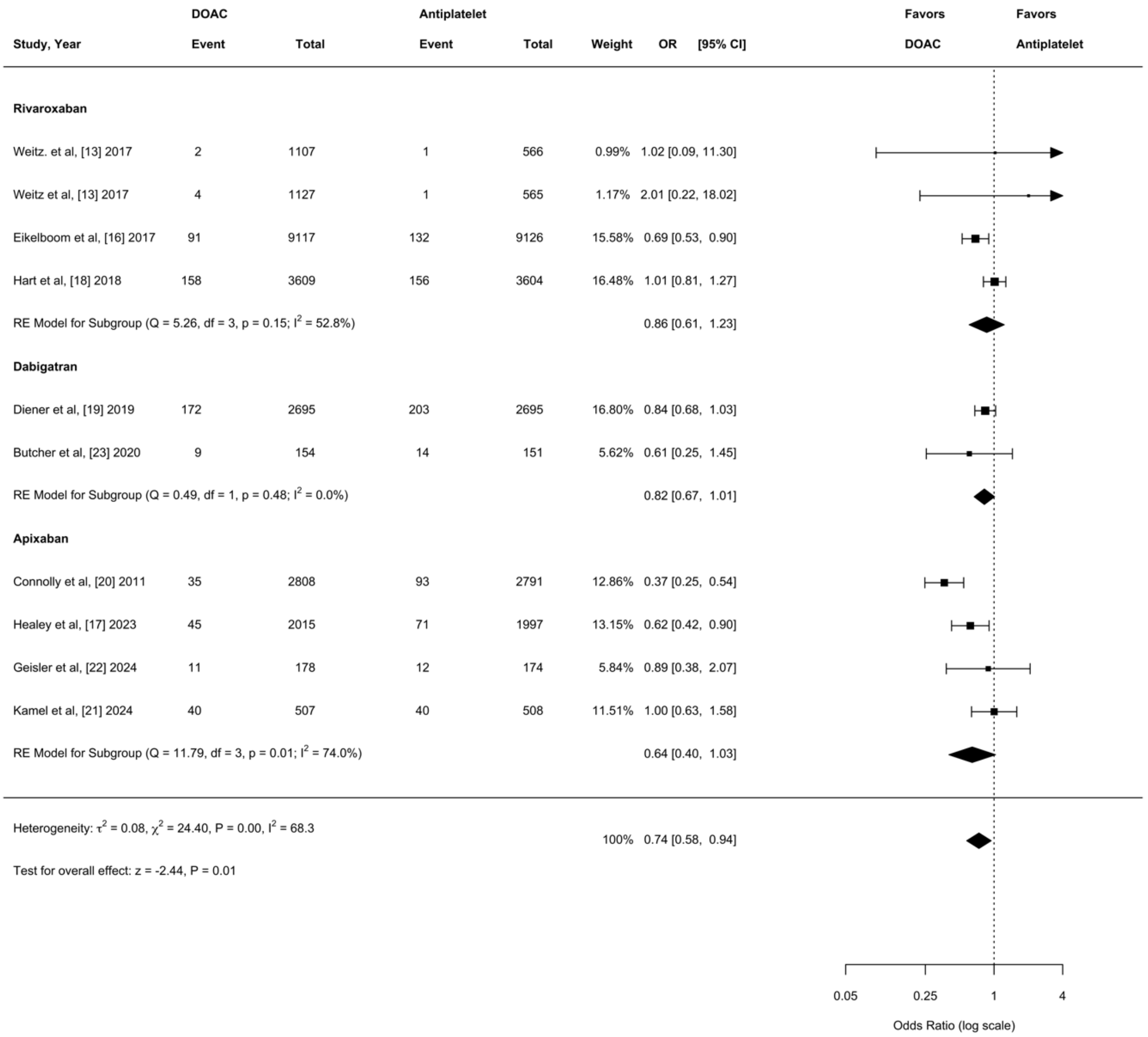

eFigure 7 – Forest plot demonstrating the association of direct oral anticoagulant therapy compared to antiplatelet therapy with ischaemic stroke. The squares and bars represent the mean values and 95% confidence intervals of the effect sizes, while the area of the squares reflects the weight of the studies. The combined effects appear as diamonds and the vertical dashed line represents the line of no effect. DOAC-Direct oral anticoagulation, CI-Confidence Interval.

© 2024 Coyle M et al. *JAMA Network Open.*

eFigure 8. Association of DOAC compared to antiplatelet therapy with CV death

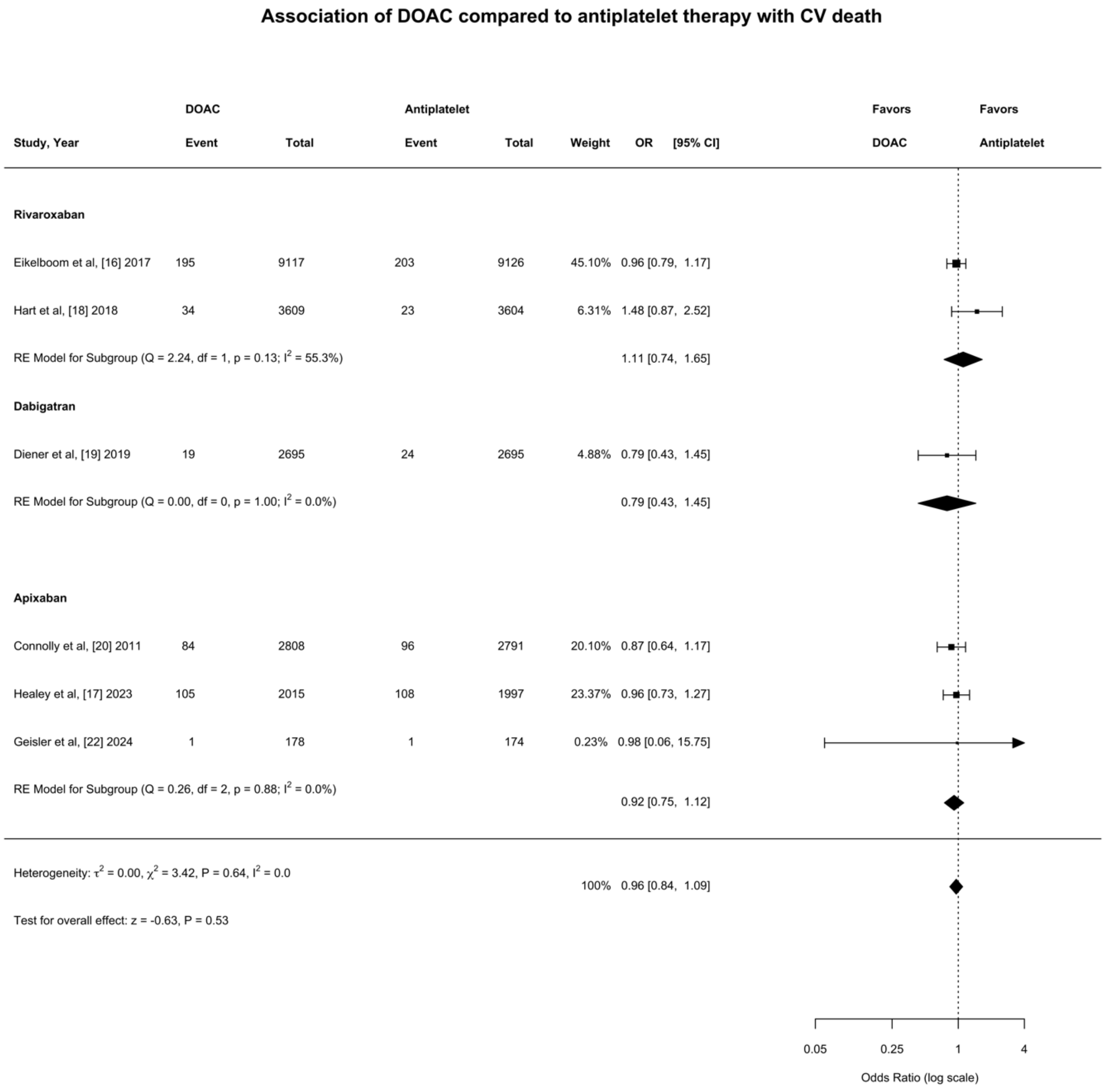

eFigure 8 – Forest plot demonstrating the association of direct oral anticoagulant therapy compared to antiplatelet therapy with cardiovascular death. The squares and bars represent the mean values and 95% confidence intervals of the effect sizes, while the area of the squares reflects the weight of the studies. The combined effects appear as diamonds and the vertical dashed line represents the line of no effect. DOAC-Direct oral anticoagulation, CI-Confidence Interval, CV-Cardiovascular death.

© 2024 Coyle M et al. *JAMA Network Open.*

eFigure 9. Association of DOAC compared to antiplatelet therapy with intracranial haemorrhage, sensitivity analysis excluding trials with zero events

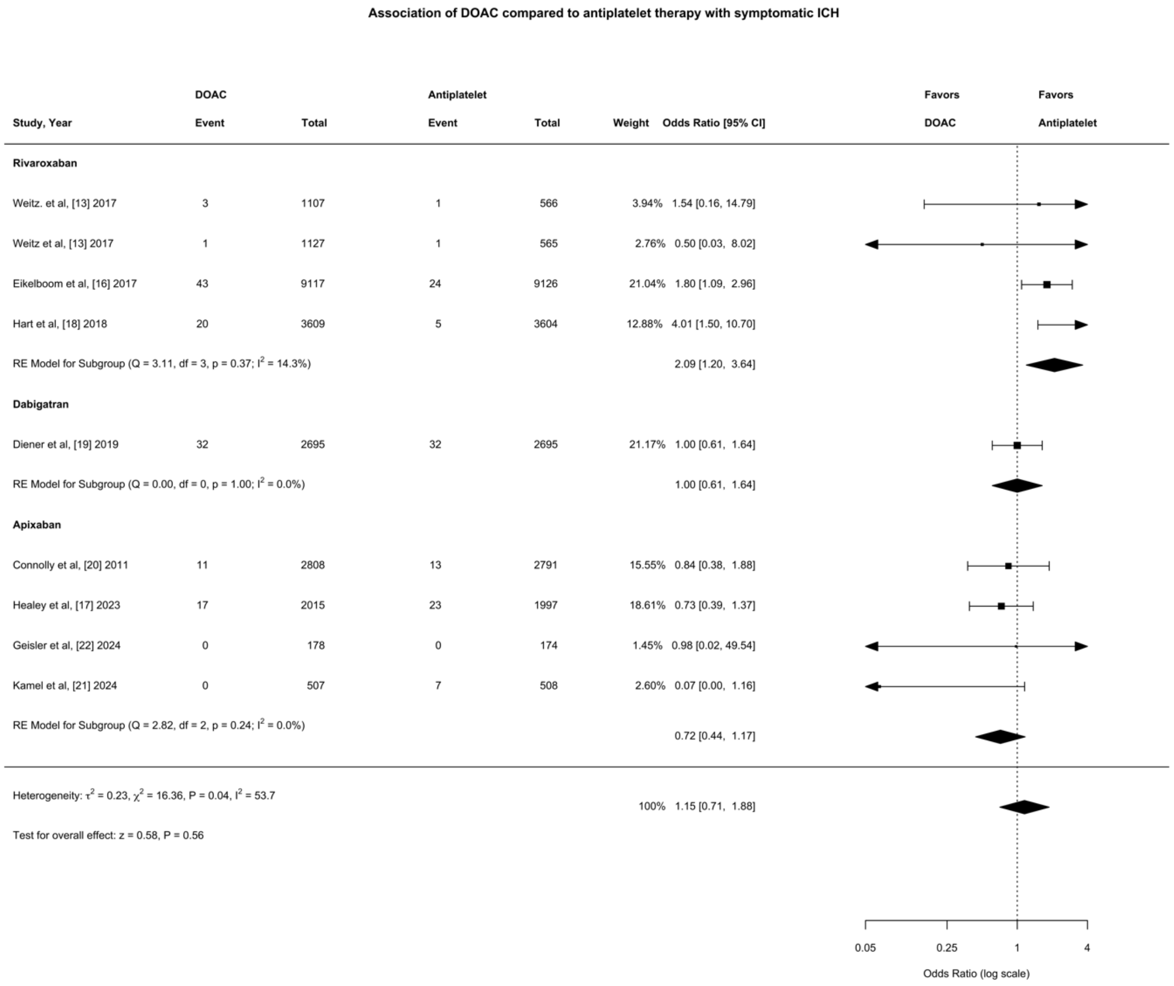

eFigure 9 – Forest plot demonstrating the association of direct oral anticoagulant therapy compared to antiplatelet therapy with symptomatic intracranial haemorrhage. The squares and bars represent the mean values and 95% confidence intervals of the effect sizes, while the area of the squares reflects the weight of the studies. The combined effects appear as diamonds and the vertical dashed line represents the line of no effect.
DOAC- Direct oral anticoagulation, CI-Confidence Interval.

© 2024 Coyle M et al. *JAMA Network Open.*
