# Supplementary material for: Risk of Intracranial Hemorrhage Associated With Direct Oral Anticoagulation vs Antiplatelet Therapy: A Systematic Review and Meta-Analysis
Source: JAMA Netw Open. 2024 Dec 4;7(12):e2449017. doi: 10.1001/jamanetworkopen.2024.49017 (PMC11618459; doi:10.1001/jamanetworkopen.2024.49017)
Supplement: Supplement 2. — Data Sharing Statement [file jamanetwopen-e2449017-s002.pdf]

Data Sharing Statement

Coyle. Risk of Intracranial Hemorrhage Associated With Direct Oral Anticoagulation vs Antiplatelet Therapy. *JAMA Netw Open*. Published December 04, 2024.
doi:10.1001/jamanetworkopen.2024.49017

Data
Data available: Yes
Data types: Other (please specify)
Additional Information: The data that support the findings of this study are available from the corresponding author upon reasonable request.
How to access data: The data that support the findings of this study are available from the corresponding author upon reasonable request.
When available: With publication

Supporting Documents
Document types: None

Additional Information
Who can access the data: Anyone requesting the data.
Types of analyses: For any purpose
Mechanisms of data availability: After approval of a proposal
